# Advances in Postharvest Storage and Preservation Strategies for *Pleurotus eryngii*

**DOI:** 10.3390/foods12051046

**Published:** 2023-03-01

**Authors:** Yuxi Guo, Xuefeng Chen, Pin Gong, Ruotong Wang, Zhuoya Qi, Zhenfang Deng, Aoyang Han, Hui Long, Jiating Wang, Wenbo Yao, Wenjuan Yang, Jing Wang, Nan Li

**Affiliations:** School of Food and Biological Engineering, Shaanxi University of Science and Technology, Xi’an 710021, China

**Keywords:** *Pleurotus eryngii*, king oyster mushroom, quality influential factors, preservation

## Abstract

The king oyster mushroom (*Pleurotus eryngii*) is a delicious edible mushroom that is highly prized for its unique flavor and excellent medicinal properties. Its enzymes, phenolic compounds and reactive oxygen species are the keys to its browning and aging and result in its loss of nutrition and flavor. However, there is a lack of reviews on the preservation of *Pl. eryngii* to summarize and compare different storage and preservation methods. This paper reviews postharvest preservation techniques, including physical and chemical methods, to better understand the mechanisms of browning and the storage effects of different preservation methods, extend the storage life of mushrooms and present future perspectives on technical aspects in the storage and preservation of *Pl. eryngii*. This will provide important research directions for the processing and product development of this mushroom.

## 1. Introduction

The king oyster mushroom (*Pleurotus eryngii*) is a high-quality, large, fleshy umbrella mushroom that is widely grown in many parts of the world [1]. It is grown in Europe, the Middle East and China [2]. *Pl. eryngii* has been intensively studied as a medicinal mushroom, a part of traditional diet and medicine, for its unique flavor, nutrition and biological functions [1]. In addition, *Pl. eryngii* has a wide market for its easily cultivated, high-yielding, and delicious product that can be cooked directly [3]. Simultaneously, *Pl. eryngii* is rich in protein, carbohydrates, unsaturated fatty acids, vitamins and other nutrients. It is also low in fat with high nutritional and medicinal value, which results in its high economic value. Its dried product contains 14.85% protein, 4.46% fat, 15.51% crude fiber, 43.15% carbohydrates and 18 amino acids. It is also rich in polysaccharides [1,4] and has good therapeutic effects, such as its activities against viruses and hypoglycemia and its ability to lower cholesterol, promote intestinal digestion, prevent cardiovascular disease and improve immunity [1,5].

Postharvest quality is a major concern for mushroom growers. *Pl. eryngii* is a highly perishable commodity and is not suitable for prolonged storage or transport over long distances [6,7]. In recent years, *Pl. eryngii* has become popular with consumers owing to its crunchy texture and nutritious nature; it meets the demand for a healthy lifestyle [8]. However, unexpected softening in texture and browning caused by polyphenol oxidase always occurs during storage, which significantly increases the challenges of postharvest storage and preservation and significantly increases the cost of transporting the king oyster mushroom [8]. From the relevant postharvest preservation studies that have been conducted on *Pl. eryngii*, drying not only has a positive impact on physical properties, such as shrinkage, dehydration capacity and color, but also on the components that exert antioxidant and health-promoting properties. These include preservation technologies, such as modified atmosphere packaging (MAP) [9,10], γ-radiation [3], 1-methylcyclopropene (1-MCP) nanopackaging and polysaccharide nanoparticle preservation [11], that can maintain their texture and nutrient content and extend the storage period. In addition, physical methods, such as microwave hot-air drying, vacuum freeze drying, solar drying and steam bleaching, can effectively reduce the loss of nutrients and reduce the intensity of respiration during storage and preservation. However, the current preservation techniques only have a small effect on the primary nutrients, and it is not known whether other nutrients are affected. Chemical methods, such as essential oils (EOs) and coating preservation [12], can delay the water loss and softening of *Pl. eryngii* to some extent and inhibit their respiration rate, thus resulting in successful storage and preservation (Table 1).

To fully preserve the nutrient contents of *Pl. eryngii*, increase its shelf life and better promote the interests of the whole king oyster mushroom industry, this paper reviews the primary manifestations of quality deterioration of these mushrooms, the quality changes of *Pl. eryngii* in postharvest storage, the mechanism of browning and the storage effects of different preservation methods, and provides a reference for the development of green preservation processes for *Pl. eryngii*.

## 2. Deterioration of the Quality of *Pl. eryngii*

The deterioration of the quality of *Pl. eryngii* after harvesting severely limits its commercial value and hinders the development of the mushroom industry. The deterioration in mushroom quality is characterized by the reduction in sensory and nutritional quality, which is owing to a combination of internal and external factors. Currently, research on the deterioration of the quality of the mushroom has focused on water loss, weight loss, postharvest morphological changes, changes in textural characteristics, color-specific changes, loss of nutrition and flavor and microbial infection.

### 2.1. Loss of Water and Weight

Fresh *Pl. eryngii* has a moisture content of up to 90% (wet basis), but its loss of moisture during storage can easily lead to weight loss, which is an important factor in the quality of fresh mushrooms [20]. A study showed that the weight loss of *Pl. eryngii* stored at 4 °C and 25 °C increased to 0.69% and 3.41%, respectively, (*p* < 0.01) compared with that of *Pl. eryngii* on day 0 throughout the storage period [21]. This is primarily owing to the exudation of cell contents, the sudden increase in the content of malondialdehyde (MDA) and the effect of related enzymes, such as superoxide dismutase (SOD), peroxidase (POD), and catalase (CAT). When the weight loss reaches 3.41% of its fresh mass, *Pl. eryngii* is considered decayed and unusable as food [21].

### 2.2. Altered Textural Properties

King oyster mushrooms are subject to aging during storage, which results in a rapid loss of hardness and contamination by microorganisms, thus explaining their short shelf life [22]. Studies have demonstrated that after 12 days of storage at 4 °C, the hardness of the mushroom decreases from 9.024 N to approximately 3.132 N. After 6 days of storage at 25 °C, the hardness of the mushroom decreases sharply to 3.11 N. Studies have shown that when the hardness of stored mushrooms decreases to less than 3.11 N, microorganisms appear on their surface, which causes them to deteriorate [21]. During storage at low temperatures, the fresh appearance of the *Pl. eryngii* is always accompanied by deterioration owing to lignification [8]. Lignification not only leads to a toughening of the *Pl. eryngii* texture and a significant reduction in nutrients but also promotes lipid peroxidation and deterioration of the king oyster mushroom substrate [8].

### 2.3. Change in Color Characteristics

Of all the quality properties that drive consumer purchasing behavior, color is the most evident dimension of quality. Among the parameters of mushroom browning, the L* value is often used to reflect the color change of the mushroom; a higher L* value indicates less browning and higher quality [21]. A study showed that the L* of *Pl. eryngii* stored at 4 °C during the first 6 days did not change significantly. The L* value of *Pl. eryngii* stored at 25 °C decreased from 94.15 to 75.33 from day 0 to day 6, respectively, indicating severe deterioration of *Pl. eryngii* [21]. When L* ≤ 82, the mushroom is of poor quality and not acceptable to the consumer. In addition to the L* value, the browning index (BI) can also be used to measure the degree of browning on the surface of king oyster mushrooms. Studies have shown that the degree of browning of the mushroom continues to increase with storage time. After 9 days of storage at 4 °C, there was slight browning on the surface of the mushroom. On day 12 of storage at 4 °C, the BI value on the surface of the mushroom reached 5.33-flod, which was no longer acceptable to the consumer compared to day 0. Compared with storage at 4 °C, the surface of the mushrooms stored at 25 °C reached a severe degree of browning after 6 days [21].

### 2.4. Loss of Nutrition

Sugars and soluble proteins in the king oyster mushroom are the primary nutrients that support ongoing metabolic activity during the postharvest phase. A reduction in protein or sugar is an important indicator of deterioration [23]. Li et al. demonstrated that the cellular oxidation of *Pl. eryngii* increased with storage time, resulting in more reactive oxygen species (ROS), which caused a decrease in reducing sugars owing to oxidation. In addition, the total free amino acid content is consumed during the pre-storage period to maintain the metabolic functions of the mushrooms. The contents of amino acids generally continue to decrease during the first 3 days of storage and do not start to increase until after 3 days. In addition to this, the fat content decreases as the storage time increases because the fat stored in the fat cells is gradually hydrolyzed by lipase into fatty acids and glycerol, which are then oxidized in other tissues [21].

## 3. Factors That Affect the Storage Quality of *Pl. eryngii*

### 3.1. Moisture

**Figure 1:**
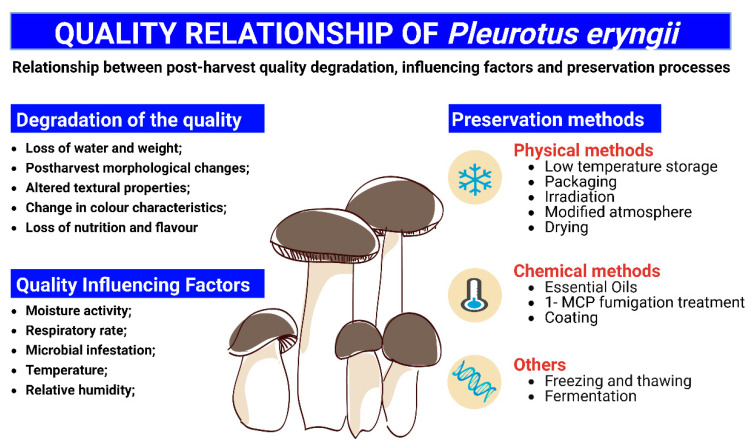
Relationship between post-harvest quality degradation, influencing factors and preservation processes of *Pl. eryngii*. Created with BioRender.com.

The tissue concentration of active polyphenol oxidase (PPO) and phenolic compounds, pH, temperature, water activity and oxygen accessibility are the most important factors that influence the rate of enzymatic browning in freshly harvested *Pl. eryngii* [4,24,25], which are highly susceptible to mechanical damage and microbial infection owing to their high contents of water (approximately 89%), lack of cuticle and presence of microorganisms on them [26]. Secondly, water loss or transpiration is an important physiological process that affects the primary quality characteristics of fresh mushrooms, such as marketable weight, appearance and texture, depending on the ambient and relative temperature and humidity [27]. Fresh *Pl. eryngii* has a very limited shelf life of 1–3 days at ambient temperature and 4–7 days at 4 °C [28]. With the increase in storage time, the apparent degradation of *Pl. eryngii* after harvesting gradually decreases in moisture, changes in internal enzymatic activity and bacterial enzymatic activity, which manifests as browning, texture softening and loss of flavor, which seriously affects its nutritional and commercial value [22] (Figure 1).

### 3.2. Respiratory Rate

The respiration rate and energy status of *Pl. eryngii* are key factors that influence postharvest senescence [29,30,31]. During storage at 25 °C, the respiratory intensity of freshly cut mushrooms increased rapidly with time, reaching 1382 CO_2_ mg/(kg·h) at 12 h and 3526 CO_2_ mg/(kg·h) at 72 h when more than 50% of the surface of the mushroom became brown and basically lost its edible value [29,30]. First, in terms of respiration rate, postharvest storage is an abiotic stress on *Pl. eryngii* since storage conditions are very different from those of growth. This storage leads to an inhibition of electron transfer in the mitochondria and an increase in the production of ROS [10]. As the levels of ROS surpass the cell’s antioxidant capacity, oxidative stress develops and mediates structural damage to lipids, membranes, proteins and DNA [32]. These results demonstrate that mitochondrial membrane enzymes implicated in mitochondrial respiratory metabolism, such as cytochrome C oxidase (CCO), will be destroyed and their function severely diminished [33,34]. To better preserve the mushrooms, the relationship between ROS and respiratory metabolism in *Pl. eryngii* is currently a hot topic in postharvest preservation research [10]. Secondly, in terms of energy metabolism, it has been shown that an inadequate supply of ATP is closely associated with a variety of postharvest symptoms, such as chilling injury, browning, yellowing and decay [35]. An adequate supply of ATP inhibits the accumulation of ROS and maintains membrane integrity, thereby delaying the aging and deterioration of *Pl. eryngii* [36]. Under postharvest abiotic stress, the energy status plays an important role in mitigating oxidative damage and maintaining organoleptic properties [36,37]. Therefore, the energy maintenance of *Pl. eryngii* during postharvest storage needs to be given high priority (Figure 1).

### 3.3. Microbial Infection

Freshly harvested *Pl. eryngii* is highly susceptible to mechanical damage and microbial infection owing to its high content of water (approximately 89%), the absence of cuticle protection and the presence of many microorganisms on its surface [4,24,25]. Decay in *Pl. eryngii* is usually induced by the tolaasin toxin in *Pseudomonas tolaasii*, which results in brown spots and yellow to dark brown lesions on the cap of the fungus [38,39,40]. In addition, other bacteria, such as *Pseudomonas azotoformans*, *Pseudomonas brenneri* and *Ewingella americana*, have been reported to be able to cause decay in *Pl. eryngii* [41]. *Listeria monocytogenes* has also been isolated from *Pl. eryngii* farm environments, which highlights the importance of monitoring the production chain from substrate production to harvesting, processing and packaging [42,43,44] (Figure 1).

### 3.4. Temperature and Relative Humidity

The various nutrients, such as polysaccharides, aldehydes and phenolic compounds, quality characteristics and microbial reproduction in the king oyster mushroom are influenced by temperature and relative humidity. Temperature fluctuations during storage can activate a variety of oxidative enzymes, enhance physiological activity, affect respiration and transpiration and increase the post-ripening period of stored *Pl. eryngii* [27], while temperature is also an important factor in determining the rate of enzymatic browning [26]. Therefore, in general, when storing *Pl. eryngii*, the shelf life is usually extended by reducing the storage temperature and increasing the ambient humidity. A storage temperature of 4–6 °C and relative humidity (RH) of approximately 95% is generally used (Figure 1).

## 4. Methods for Storing and Preserving *Pl. eryngii*

### 4.1. Physical Methods and Mechanism

#### 4.1.1. Modified Atmosphere Packaging (MAP)

**Figure 2:**
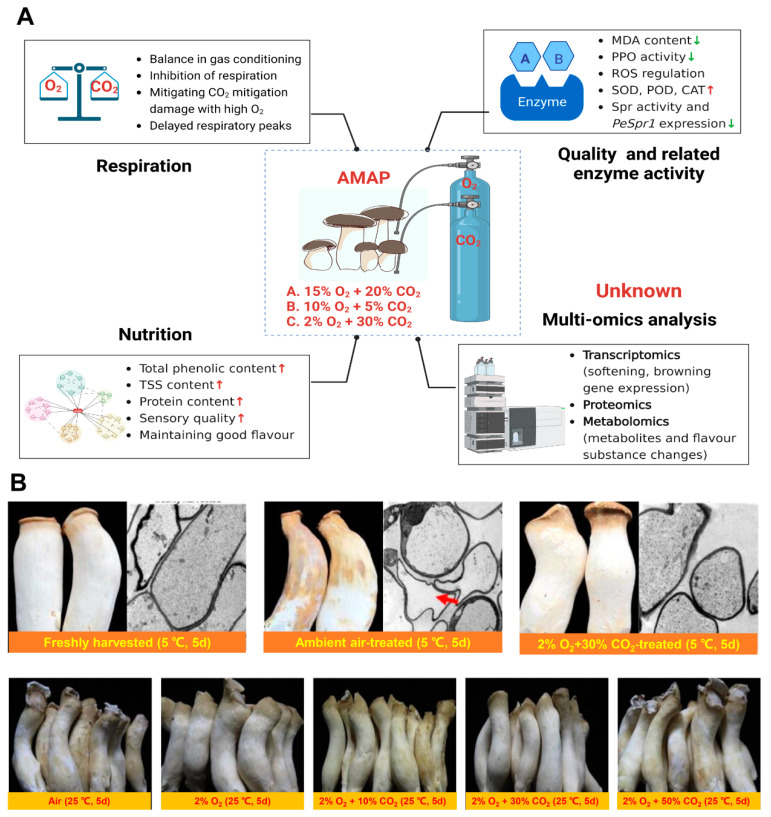
Mechanism of action of MAP preservation of *Pl. eryngii* (**A**) and changes in *Pl. eryngii* morphology from existing MAP research (**B**). (**A**) Created with BioRender.com. (**B**) cited from [9,10] The arrow in the figure points to the destruction of the cell structure. ©Copyright 2012, Elsevier. ©Copyright 2015, Elsevier.

MAP is used to control the proportion of nitrogen, oxygen, carbon dioxide and ethylene in the gas, humidity, temperature (above the freezing threshold) and air pressure of the gas in the gas conditioning warehouse, thereby inhibiting the amount of cellular respiration and reducing the metabolic rate, so that the mushrooms are nearly dormant, thus preserving them over a long-term period [45]. As a complement to storage temperature control, MAP has been found to be a simple, economical and effective postharvest preservation technique for commodities [46]. Four core parameters need to be considered when designing MAP, including the product characteristics, the permeability of the packaging material, gas concentration (carbon dioxide and oxygen) and temperature dependence [47,48]. The quality of the MAP of *Pl. eryngii* is related to texture, microbial count, whiteness, color variation and organoleptic characteristics, which are essential for the analysis of spoilage rates and thus influence consumer acceptance [49]. Figure 2 summarizes the mechanism of action of MAP preservation and the changes in *Pl. eryngii* morphology from existing MAP studies.

To investigate the total quality index of king oyster mushrooms treated with different gas mixtures of MAP after harvesting, Wan-Mohtar et al. [13] investigated this and showed that high CO_2_ packaging (HCP) (20% CO_2_ and 15% O_2_) retained the best qualities of king oyster mushrooms. HCP recorded the highest total phenolic content (TPC) and showed the highest effectiveness in maintaining the color and odor of *Pl. eryngii* compared with the control and low CO_2_ packaging (LCP: 2% CO_2_ and 30% O_2_). Briones et al. [50] suggested that the use of 2.5–5% CO_2_ and 5–10% O_2_ would result in optimal storage conditions for mushrooms. For safety reasons, it is recommended that O_2_ should not be less than 2% under MAP conditions [51]. Research by Jafri et al. [52] that utilized 10% O_2_ + 5% CO_2_ for the MAP treatment of king oyster mushrooms showed that this model was more effective at retaining quality characteristics and higher organoleptic ratings compared with other samples, which could be maintained for a storage period of 25 days. The treated mushrooms showed minimal changes in weight loss, pH and total soluble solids. Free radical scavenging activity and the total polyphenol contents were maintained at 85% and 91%, respectively [52]. The effect of MAP on the enzymatic activity and shelf life of king oyster mushrooms stored at 20–25 °C and 90–95% RH for 5 days was investigated by Li et al. The results indicated that 2% O_2_ + 30% CO_2_ significantly prolonged the shelf life of the mushrooms compared with the control. A total of 2% O_2_ + 30% CO_2_ mixture was more suitable for maintaining the organoleptic properties of the mushrooms and delaying the increase in MDA and O_2_ production during storage. In addition, the activities of SOD, POD and CAT were significantly higher than those of the control. Treatment with 2% O_2_ + 30% CO_2_ reduced lipid peroxidation and enhanced the activity of antioxidant enzymes but had little effect on the CCO activity of the mushrooms [10].

The molecular mechanisms of postharvest senescence also merit attention. Zhang et al. [9] showed that the shelf-life of the mushrooms was prolonged after 2% O_2_ + 30% CO_2_ treatment and that the cell morphology was normal with no obvious aberrations, and the cytoplasmic distribution was as uniform as that of freshly harvested mushrooms, which significantly inhibited cell abnormalities, serine protease activity and *PeSpr1* expression. However, there is a lack of research on the flavor and nutrient changes caused by metabolic substances during the gas conditioning process of king oyster mushrooms, which is crucial for acceptability by consumers. Further studies on the transcriptome, proteome, metabolome and multi-omics of this mushroom after gas conditioning treatment should be strengthened to provide a theoretical basis for the gas conditioning preservation mechanism (Figure 2).

#### 4.1.2. Special Packaging

**Figure 3:**
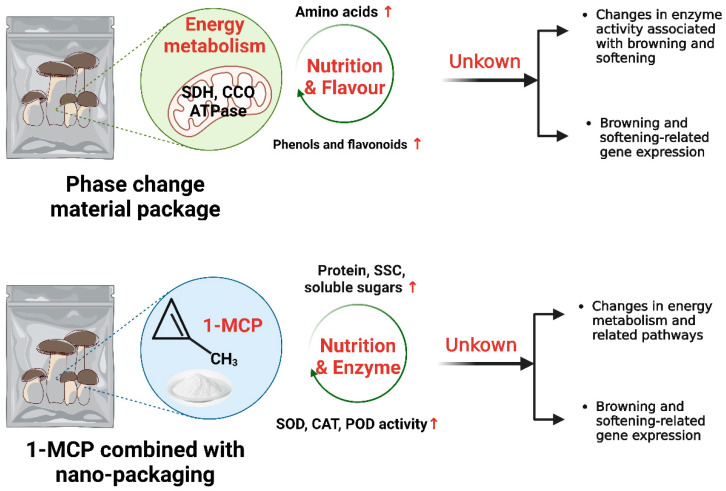
Mechanism of action of special packaging preservation of *Pl. eryngii*. Created with BioRender.com.

Phase change materials (PCMs) are substances that absorb latent heat through phase changes and play an important role in short-duration cold chain transport [53]. Li et al. [14] developed a new water-based PCM and showed that king oyster mushrooms treated with the new PCM accumulated the most phenolics and flavonoids in all three groups, which mitigated the deterioration of its appearance during storage (Figure 3). The measurements of free amino acids demonstrated that the new PCM treatment increased the levels of phenylalanine, glutamic acid (Glu) and proline (Pro) by creating low-temperature conditions, thus improving the nutritional quality and flavor attributes and delaying the postharvest aging of king oyster mushrooms. In addition, the new PCM treatment maintained an adequate energy supply to the mushroom by activating the activities of succinate dehydrogenase, CCO and ATPases, thus reducing the catabolism of Pro and Glu.

The application of nano-packaging can extend the life of postharvest edible mushrooms and maintain their original color and taste [54,55]. 1-MCP, a type of cyclopropene, has been widely used and shown to inhibit the action of ethylene in respiratory senescent fruit by competitively binding to ethylene receptors [56]. Xu et al. indicated that 1-MCP combined with nanopackaging treatment was effective at suppressing the increase in respiratory intensity, weight loss, MDA content and PPO activity of *Pl. eryngii* at 4 °C, delaying the decrease in soluble protein content, maintaining soluble sugar and soluble solid content and increasing the activities of SOD and POD, thereby maintaining the postharvest quality of king oyster mushrooms and extending the storage time [11]. The efficiency of the combined treatment was superior to that of the sole packaging with 1-MCP or nano compared with the untreated samples [11].

Currently, nanopackaging studies on Enoki mushrooms (*Flammulina velutipe*) are relatively thorough [57,58,59,60] and complete in terms of basic physicochemical indicators, reactive oxygen metabolism, energy metabolism, proteomics and metabolomics to elaborate the storage quality, primarily browning and softening, of Enoki mushrooms extreme mechanisms of action. The study on king oyster mushrooms can also be studied in-depth in this respect in terms of a single packaging technique, which expands its intrinsic preservation mechanisms in terms of energy and multi-omics expression.

#### 4.1.3. Low-Temperature Storage

**Figure 4:**
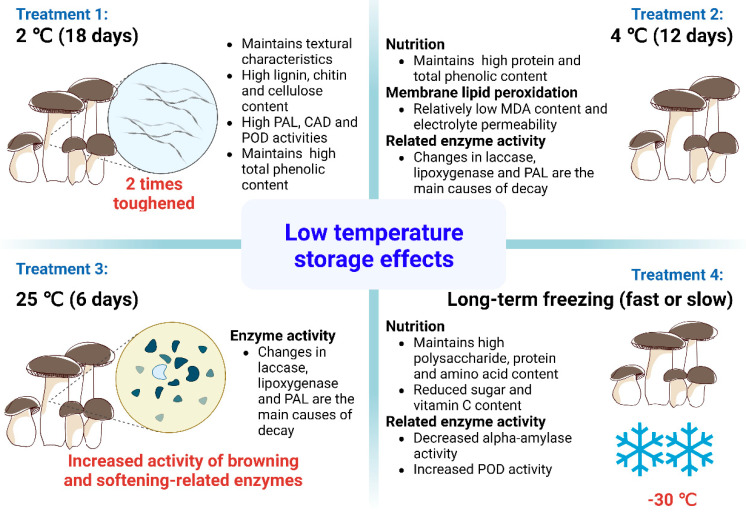
Mechanism of action of *Pl. eryngii* for low-temperature storage and preservation. Created with BioRender.com.

Low-temperature storage is a common way to store and preserve edible mushrooms. Low temperatures can inhibit enzyme activity, reduce physiological metabolic activity, reduce the respiratory intensity and inhibit the growth and reproduction of microorganisms (Figure 4). Li et al. [8] conducted a related study on this in 2015 and showed that the optimal treatment was 2 °C and that toughening occurred twice throughout the storage process. This treatment maintained high textural properties for 18 days, with higher contents of chitin and higher activities of phenylalanine ammonia lyase (PAL), CAT and POD, and maintained a high content of total phenolics and lower membrane lipid peroxidation. This also suggests that toughening may be primarily caused by oxidation and can affect the quality of the mushrooms after harvesting [8]. A further complementary study on the same preservation method by Li et al. in 2021 compared quality parameters, chemical composition, MDA concentration and metabolic enzyme activity during storage at 4 °C for 12 days and at 25 °C for 6 days. The best treatment measure was found to be the treatment group stored at 4 °C for 12 days, which maintained high quality, high nutritional characteristics, a high content of total phenolics, progressively higher enzyme activity and low membrane lipid peroxidation. Simultaneously, increased activities of laccase, lipoxygenase and PAL and the accumulation of MDA, as well as polysaccharide degradation, were the primary factors that contributed to the deterioration of the king oyster mushrooms during storage [21].

Freezing prevents the growth of microorganisms and preserves the texture of tissues and the nutritional value of food [61]. Long-term freezing (fast or slow) is the appropriate way to preserve mushrooms for the long term [61]. It involves the extensive exposure of cells to low temperatures and dehydration. Jiang et al. [62] evaluated the metabolite content of substrates to improve the understanding of changes in the nutritional composition of king oyster mushrooms during short-term slow frozen storage. The study showed that the optimal treatment was a storage temperature of −30 °C for the caps, which maintained a high nutritional value. The content of polysaccharides, proteins and amino acids in the cap increased and then decreased, while the content of all measured substances in the stalk slowly decreased. The activity of α-amylase decreased; that of POD increased, and the contents of reducing sugars and vitamin C continuously decreased with the extension of the freezing time [62].

#### 4.1.4. Irradiation

**Figure 5:**
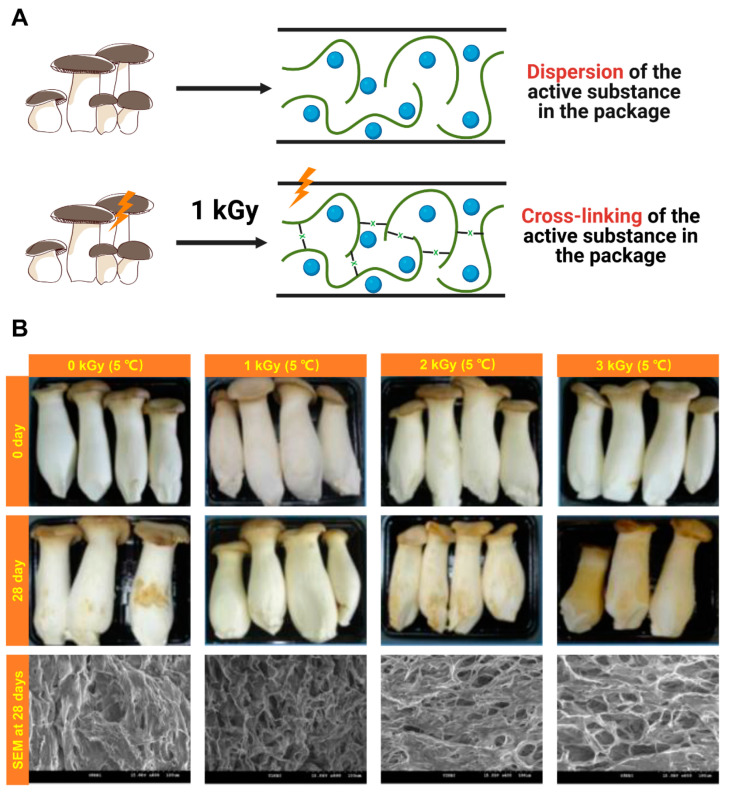
Mechanism of action of irradiation preservation of *Pl. eryngii* (**A**) and changes in *Pl. eryngii* morphology and SEM images from existing irradiation research (**B**). (**A**) Created with BioRender.com. (**B**) Cited from [3] ©Copyright 2012, Elsevier.

The application of improved postharvest techniques, such as food irradiation, can improve marketability and extend storage life, and the technology is now widely commercialized [63,64]. The technical suitability and nutritional safety of irradiated foods have been well studied [31,64]. Low doses of irradiation of fresh produce can provide hygienic safety and affect different physiological processes, such as enzyme activity and respiration, thus significantly improving postharvest storage [22,65,66]. Akram et al. investigated the quality attributes of irradiated king oyster mushrooms. The study showed that the best treatment measure was irradiation at 1 kGy and that the L-value (brightness) of this group increased after irradiation and remained high throughout storage, maintaining a good appearance as indicated by homogeneous color and the absence of fungal decay and blemishes, good hardness and microstructure and low weight loss [3]. Irradiation at 1 kGy was the most effective for extended postharvest storage and had additional advantages [3] (Figure 5). However, irradiation treatment requires a high level of skill on the part of the operator and still requires significant consideration of its cost.

#### 4.1.5. Drying

Drying is a typical approach to food preservation based on the principle that the water activity of the product should be minimized to a defined level to ensure microbiological and physicochemical stabilization; it has been used for many years to improve the shelf life of food commodities [67,68]. Hot blanching is receiving increasing attention as a pretreatment method to improve drying quality [69]. The current scalding process, which is conducted by direct interaction between the sample and a medium, such as hot water and steam [70], can significantly (*p* < 0.05) reduce the total number of bacteria, improve drying efficiency and reduce the level of browning of the sample during drying [71]. However, shortcomings of water and steam blanching have been reported, including the loss of nutrients, such as vitamins, proteins and polysaccharides, and uneven blanching [70]. The results of Tolera et al. showed that the optimal treatment was a solar drying method with an infiltration concentration of 5%, which reduced the moisture by 7.74% and maintained the following proximal component contents: crude protein content 25.13% db, crude fat 2.27% db, total ash 10.17% db, crude fiber 10.26% db and carbohydrates 44.42% db. The purpose of microwave hot-air flow rolling dry-blanching (MARDB) pretreatment is to improve the drying efficiency and quality of the king oyster mushroom [72]. Microwaving can alter the microstructure during the drying-hot blanching process, which could affect the drying characteristics, water state and migration [72]. Su et al. [15] revealed that optimal pretreatment (9 min) with MARDB significantly improved the quality indicators, such as color, water content and polysaccharide content of *Pl. eryngii*, shortened the drying time and completely deactivated PPO and POD. T2 relaxation spectra and microstructural analysis indicated that the primary reason for the improved drying efficiency at the optimal MARDB time was the resistance to free water migration and reduction in the pore structure. Excessive hot blanching (12 min) prolongs the drying time and leads to a reduction in whiteness and the contents of polysaccharides and phenolics [15]. Ucar et al. [73] freeze-dried *Pl. eryngii* at −20 °C, which maintained a better color and preserved the textural properties to prevent softening. However, the cost is relatively high when it comes to industrial production [73].

### 4.2. Chemical Methods and Mechanism

#### 4.2.1. Essential Oil Treatment

EOs are natural volatiles obtained by distillation and have the characteristic aroma of the plants from which they are extracted [74]. An EO acts on the biochemical processes of the mushroom and inhibits or increases the concentration of enzymes and secondary metabolites associated with the preservation of quality [75,76]. Manjari et al. [77] conducted an experiment to study the effect of different essential oils on the enzymatic activity of stored *Pl. eryngii*. The results showed that the best treatment was peppermint oil (10 μL), which maintained high contents of total phenolics, TPC (0.286 mg/g), PAL (0.038 μM/g), PPO (0.042 U/mg) and POD (0.38 U/mg). The higher levels of TPC and PAL in the *Pl. eryngii* treated with EO and the lower levels of PPO and POD in the treated samples compared with those of the control indicated that the EO treatment had a positive effect on the quality of the harvested mushrooms [77]. This preservative technique will help to extend the shelf life of the harvested substrates. Studies have reported that EOs have a significant antibacterial effect [78], but there is a lack of available research on the antibacterial effect and mechanism of action of EOs on *Pl. eryngii* during storage.

#### 4.2.2. Coating

In recent years, many different types of edible coatings have been successfully explored and further developed for the postharvest storage of mushrooms [79]. Chitosan is a biodegradable polymer that occurs naturally and can be applied as an edible coating to suppress changes in the quality of mushrooms during storage [80]. Liu et al. [12] investigated a solution of protocatechuic acid grafted chitosan (PA-g-CS) with an antioxidant potential as a possible new edible coating material for the postharvest storage of king oyster mushrooms [12]. The results showed that the best treatment was the PA-g-CS III (high grafting rate) coating group, which was able to maintain good textural properties, low membrane lipid peroxidation, high activities of SOD, ascorbate peroxidase (APX), glutathione reductase (GR) and CAT and low activity of PPO [12].

**Figure 6:**
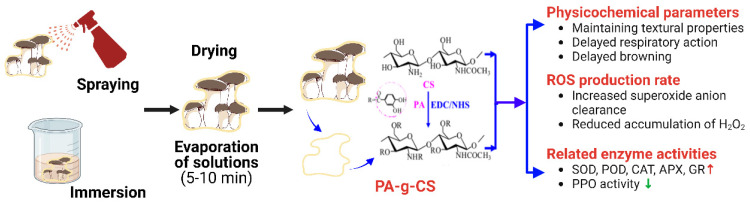
Mechanism of action of *Pl. eryngii* for coating. Created with BioRender.com.

There is good current acceptance of edible coating films in mushroom preservation, but there is still a need to expand the use of edible coating solutions of natural plant origin in king oyster mushrooms. Moreover, the mechanism of action of coated film preservation in *Pl. eryngii* merits further study, such as the use of a multi-omics approach to elucidate the expression of relevant browning and softening genes, protein up-/downregulation and flavor changes during storage caused by differential metabolites. In addition to this, changes in energy owing to respiration after film coating for preservation need to be considered to elucidate the mechanisms of preservation (Figure 6).

### 4.3. Others

#### 4.3.1. Different Freeze–Thaw Treatments

In contrast to slow block freezing, the single-piece quick freezing method uses cryogenic gases to rapidly reduce the temperature of mushrooms to the freezing point, which maintains cellular integrity with little change in nutritional quality and organoleptic properties [81]. In general, frozen foods need to be thawed before processing and consumption, and thawing has a direct or indirect effect on the quality of the product. Therefore, freezing and defrosting are equally important to consumers. There are several methods of defrosting that are frequently used by consumers, such as natural air convection defrosting (NT), flow-through defrosting (FT) or microwave defrosting (MT) [81]. Li et al. used the natural freezing (NF, −20 °C) or single freezing (−62.5 °C, speed 8.23 m/s) methods to freeze cut king oyster mushrooms, and three thawing methods, including flowing water (FT, 4 °C), microwaving (MT, 620 W) and natural air convection (NT, 20 ± 5 °C), to thaw the mushrooms [17]. The results of the study showed that the best treatment measure was individual quick freezing and thawing with NT at room temperature, which was able to maintain cell integrity, preserve the texture of king oyster mushrooms and maintain high water holding capacity, low thawing losses, good color and good flavor. As a result, the method minimizes changes in the quality of frozen king oyster mushrooms [17].

#### 4.3.2. Fermentation

As one of the oldest processing techniques, lactic acid fermentation is recognized as a highly valuable processing approach to retain and improve the safety, nutritional and sensory characteristics of vegetables [82]. In addition, varieties of lacto-fermented vegetables are often classified by their composition and method of preparation. For example, sauerkraut, kimchi, such as that made from cucumber and olive, and kimchi are the most investigated lacto-fermented vegetables, predominantly for their commercial importance [83,84]. Today, pure fermentations of lactic acid bacteria (LAB) are widely used on a commercial scale for these commodities. As a result, this technique offers advantages over traditional methods, including shortened fermentation cycles, the elimination of non-lactic acid contaminants, and rapid fermentation at higher temperatures. It also ensures hygienic conditions and maintains consistency for better quality and flavor. *Lactobacillus plantarum* is an important member of the LAB family and is commonly used to ferment vegetables [85]. Zheng et al. studied the preservation of king oyster mushrooms using three typical lactic acid fermentation processes, including sauerkraut, pickling and kimchi, with *L. plantarum* as the fermentation agent. This study showed that controlling the heavy salt pickling process inhibited microbial growth and reproduction and rendered most microorganisms inactive. These LAB rapidly colonize the mushroom substrate and quickly control spoilage and pathogenic microorganisms [16]. The final fermentation product contained high levels of LAB (>7 Log CFU/g). In addition, the nitrite concentration in the final fermentation product was below the current maximum level permitted in China (<20 mg/kg). The results indicate that the lactic acid fermentation method is effective and safe for the preservation of king oyster mushrooms [16].

#### 4.3.3. Polysaccharide Nanoparticle Preservation

Chitosan nanoparticles are used to encapsulate bioactive substances owing to their good biocompatibility, high efficiency of encapsulation, safety and non-toxic properties [86]. Therefore, if chitosan-based nanoparticles are used in combination with antimicrobial agents, such nanoparticles may induce synergistic effects between chitosan and antimicrobial agents [87]. Microbial contamination usually occurs on the surface of food products. When nanoparticles are sprayed directly onto the food surface, vesicles and uneven distribution can occur, thus weakening the antimicrobial effect. The morphological transformation from nanoparticles to nanofibers is considered a feasible approach because nanofibers have a larger specific surface area and disperse more effectively. Pomegranate peel polyphenol (PPP), a natural, safe and green antimicrobial agent, was introduced and embedded in chitosan to form stable nanoparticles. PPP chitosan nanoparticles (PPP-CNPs) were further electrospun into king oyster mushroom polysaccharide (PEP)-based nanofibers. The optimal treatment measure of PPP 3 mg/mL was obtained by Cai et al. [19]. This group was able to maintain small nanoparticle size and uniform nanoparticle dispersion, maintain optimum stability, produce tighter nanofibers, improve the thermal stability of PEP nanofibers, inhibit the activity of *E. coli* O157: H7 on the food surface, maintain good color quality and obtain the highest encapsulation rate of 23.71 ± 0.51% [19]. However, the safety of this type of preservation technology in industrial applications that produce king oyster mushrooms still requires additional evaluation.

## 5. Challenges and Future Trends

There are currently relatively few effective means to commercially preserve *Pl. eryngii*. They primarily include low-temperature storage, gas preservation and vacuum drying, and their ability to preserve the mushrooms is highly inadequate for the needs of industrialization. Therefore, it is important to study the mechanisms that cause the quality of *Pl. eryngii* to deteriorate and implement new preservation techniques to extend the shelf life of these mushrooms. Based on the mechanism of the deterioration in the quality of king oyster mushrooms, future research should focus on the following aspects.

(1) To further investigate the mechanisms of quality fission during storage and preservation, such as browning, softening and lignification, and to use multi-omics techniques to study the potential molecular mechanisms of gene regulation in different preservation methods. This approach should help to address the problem of postharvest quality deterioration of king oyster mushroom strains at the molecular level.
(2) Research on the mechanisms of nutrient retention and flavor transfer during storage and the effects of different preservation methods on the biological activity and quality characteristics of king oyster mushrooms should be strengthened to improve the quality characteristics of king oyster mushrooms after preservation while extending its shelf life and greatly enhancing its commercial value.
(3) Among the methods of preserving *Pl. eryngii*, relatively little research has been conducted on the use of radiation, ozone and film coatings to preserve these mushrooms. There is still a need to explore the effects of these traditional methods of preserving edible mushrooms on *Pl. eryngii* and the mechanism of preservation, as well as the development of new green preservatives, based on natural types of bioactive substances.
(4) In the future, a combination of new and traditional technologies can be used to improve the postharvest quality of *Pl. eryngii*, such as combining radiation treatment with 1-MCP in concert with nanopackaging treatment, developing cold sterilization equipment, creating safe and efficient sterilization processes, such as irradiation, microwave, low-pressure electrostatic field and low-temperature plasma sterilization equipment and processes, and decreasing the deterioration of the quality of *Pl. eryngii* during storage and distribution.

## Figures and Tables

**Table 1:** Methods of *Pl. eryngii* storage and preservation.

Treatments	Process Parameters	Storage Days	Preservation Effect	Ref.
Modified atmosphere packaging	Storage temperature: 4 °C Storage relative humidity: 95% Grouping processing: -high carbon dioxide packaging (HCP: 20% CO_2_ + 15% O_2_) -low carbon dioxide packaging (LCP: 30% O_2_ + 2% CO_2_) -high nitrogen packaging (HNP: 85% N_2_, 15% O_2_)	10 d	Optimal processing: HCP: 20% CO_2_ + 15% O_2_ -High total phenolic content -Darkening delaying effect	[13]
High carbon dioxide and low oxygen storage	Storage temperature: 4 °C Storage relative humidity: 95% Grouping processing: -2% O_2_ + 30% CO_2_ -Air	5 d	Optimal processing: 2% O_2_ + 30% CO_2_ -Inhibition of serine protease activity	[9]
	Storage temperature:4 °C Storage relative humidity:95% Grouping processing: -2% O_2_ -2% O_2_ + 10% CO_2_ -2% O_2_ + 30% CO_2_ -1% O_2_ + 50% CO_2_ -Air	5 d	Optimal processing: 2% O_2_ + 30% CO_2_ -O_2_^−^ production rate: 50.7% -Improve enzyme activity (SOD)	[10]
1-MCP treatment combined with nano-packaging	Storage temperature:4 ± 1 °C Storage relative humidity: 90–95% Grouping processing: -Untreated -1-MCP (0.3 μL L^–1^, 24 h) -Nano-packaging -1-MCP (0.3 μL L^–1^, 24 h) + nano-packaging.	12 d	Optimal processing: 1-MCP + nanopackaging -Texture enhancement -Delay respiration rate -Soluble protein improved -Avoid the accumulation of activated oxygen and enhance antioxidant activity (PPO, SOD and CAT)	[11]
A novel phase change material	Storage temperature: 22 °C ± 2 °C Preparation of PCM: 0.01% nano-TiO_2_, 2.09% K_2_SO_4_, 1.72% maltitol, and 0.50% superabsorbent polymer Grouping processing: -Novel PCM (−2 °C) -Ice (−2 °C) -Equal mass of water	5 d	Optimal processing: the novel PCM (−2 °C) -Total flavonoid contents: 37.31% higher than control -Free amino acids: the contents of Glu, Phe and Pro were 1.95-fold, 1.34-fold and 2.07-fold higher than those in control, respectively; electrolyte leakage: 17.94% lower than that in control -Antioxidant activity enhancement (GDH, POD, SOD and CCO)	[14]
Gamma irradiation	Storage temperature: 5 ± 1 °C Group: 0, 1, 2, 3 kGy	28 d	Optimal processing: 1 kGy -Uniform color with no fungus spoilage and blemishes -Scanning electron microscopy: comparable micro-structure to that of the control	[3]
MARDB (microwave hot-air flow rolling dry-blanching)	Storage temperature: 4 °C MARDB pretreatment: constant microwave power: 3 W/g, the speed of the rolling bed: 5 rpm Hot-air drying treatment: speed of rolling bed: 5 rpm, drying temperature of the material: 60 °C Group processing: -After pretreatment, cooled to 60 °C in the air and dried. -After pretreatment, packed in plastic bags, sealed and placed in the refrigerator of 4 °C	12 d	Optimal processing: microwave hot-air flow rolling dry-blanching for 9 min -Maintaining quality parameters -Maintain moisture ratio -Reducing water holding capacity and water binding capacity	[15]
Temperature-controlled cold rooms	Relative humidity: 87 ± 5% Packing material: PE Group: 2 °C low temperature 4 °C low temperature 8 °C low temperature	18 d	Optimal processing: 2 °C low temperature -High total phenolic content -Darkening delaying effect -Membrane lipid peroxidation is low	[8]
Distilled water coating, CS coating, PA-g-CS I (low grafting 125degree) coating, PA-g-CS II (medium grafting degree) coating, PA-g-CS III (high grafting degree) coating	Treatment Time: 30 s Storage temperature: 4 ± 1 °C Relative humidity: 95% Group: -Control (distilled water coating) group -CS coating group -PA-g-CS I (low grafting degree) coating group -PA-g-CS II (medium grafting degree) coating group -PA-g-CS III (high grafting degree) coating group	15 d	Optimal processing: PA-g-CS III (high grafting rate) coating group -Maintain high quality -Lower membrane lipid peroxidation -Antioxidant activity enhancement (SOD, APX, GR, CAT) -Microstructure: PA-g-CS coating group has a less entangled fiber structure and smaller pores.	[12]
Lactic acid fermentation	Group: -Storage temperature: 20 °C Sauerkraut process: 2% salt, 1% crystal sugar, and 0.1% Lactic Acid Bacteria Powder Starter -Storage temperature: 4 °C Kimchi process: 4% solar salt, 2% sugar and 0.1% Lactic Acid Bacteria Powder Starter -Storage temperature: 30 °C Pickle process: 50 mM acetic acid, 2.06 M NaCl and 2% sugar and 0.1% Lactic Acid Bacteria Powder Starter -Storage temperature: 20–25 °C Control heavy salting process: Saturated brine (450 mL, 25%, approximately)	30 d	Optimal processing: Control heavy salting process -Microbial counts changes: no count of lactic acid bacteria and Enterobacterial was detected; yeasts and molds were able to survive at 30 days -Inhibit the action of microorganisms: pH and titratable acidity: nearly unchanged -Nitrite concentration: relatively low and stable	[16]
Natural freezing (NF, −20 °C) or individually quick-frozen (IQF) (−62.5 °C and speed 8.23 m/s) methods	Storage temperature: −20 °C Group: -NF, thawed by NT at room temperature -NF, thawed by FT at 4 °C -NF, thawed by MT at 620 W. -IQF, thawed by NT at room temperature -IQF, thawed by FT at 4 °C -IQF, thawed by MT at 620 W	—	Optimal processing: IQF, thawed by NT at room temperature -Thawing curve: takes less time to reach 4 °C -Water holding capacity: significantly higher than that of NF; thawing loss: significantly lower than that of NF -Cutting force analysis: high hardness -Sensory evaluation of thawed mushroom: superior to NF samples in all aspects; IQF least affected the quality after thawing	[17]
freezing or canning	Group: Storage temperature: −25 °C Freezing and Canning	—	Optimal processing: Boletus edulis, Freezing Preservation effect: -The coefficients for converting total nitrogen to protein: 4.18	[18]
PPP@chitosan nanoparticles	Storage temperature: 37 °C Group: -PPP 1.5 mg/mL -PPP 3 mg/mL -PPP 4.5 mg/mL -PPP 6 mg/mL	5 d	Optimal processing: PPP 3 mg/mL -Inhibit the activity of *E. coli* O157:H7 on food surfaces. Antimicrobial activity: pork: The number of *E. coli* O157:H7 decreased by 99.02% and 99.11% cucumber: the number of *E. coli* O157:H7 decreased by 99.48% and 99.77%	[19]

## Data Availability

Not applicable.
